# Posner-Schlossman Syndrome in Common Variable Immunodeficiency

**DOI:** 10.1155/2020/8843586

**Published:** 2020-10-15

**Authors:** Madiha Huq, Neha Sanan, Phuong Daniels, Robert Hostoffer

**Affiliations:** ^1^University Hospitals Parma Medical Center, Cleveland, Ohio, USA; ^2^University Hospitals, Cleveland Medical Center, Cleveland, Ohio, USA; ^3^Lake Erie College of Osteopathic Medicine, Erie, Pennsylvania, USA; ^4^Allergy/Immunology Associates Inc., Mayfield Heights, Ohio, USA

## Abstract

**Introduction:**

Posner-Schlossman syndrome (PSS) is a rare glaucomatocyclitic crisis with clinical features including recurrent episodes of unilateral elevated intraocular pressure. Autoimmune and infectious causes have been proposed as potential etiologies of PSS. We report the first case of PSS in the setting of common variable immunodeficiency (CVID). *Case Report*. A sixty-two-year-old Caucasian female with a medical history of CVID and ulcerative colitis presented to the emergency room with complaints of acute right-sided vision changes. She reported image distortion, blurriness, and loss of central vision. Physical exam was significant for mildly injected right conjunctiva, visual acuity of 20/70 in right eye, and 20/25 in left eye. The right intraocular pressure was measured at 34 mmHg and left at 12 mmHg. The gonioscopy and dilated fundus examination were unremarkable. Cup to disc ratio was within normal limits, and no afferent pupillary defects were recorded. The patient was acutely treated with three rounds of dorzolamide/timolol and 0.2% brimonidine which decreased the right eye intraocular pressure to 24 mmHg. On follow-up exam with an ophthalmologist, anterior uveitis including an elevated pressure of 41 mmHg on the right and 18 mmHg on the left eye was noted and a PSS diagnosis was confirmed.

**Conclusion:**

PSS remains a rare condition with uncertain etiology and no associated systemic conditions. PSS has been postulated to be linked to autoimmune conditions. CVID is associated with many autoimmune disorders including Sjogren's, rheumatoid arthritis, and colitis. There have been a few reported CVID-associated ocular diseases including granulomatous uveitis and conjunctivitis, chronic anterior uveitis, and birdshot retinopathy. We describe the first case of PSS in a patient with CVID.

## 1. Introduction

Posner-Schlossman syndrome (PSS), a glaucomatocyclitic crisis with unknown etiology, was first reported in 1948. Common clinical features include recurrent episodes of unilateral markedly elevated intraocular pressure (IOP) that is associated with mild anterior uveitis. Each attack can last a few hours to a month and usually affects middle-aged adults [1, 2]. PSS typically presents with mild discomfort, halos around lights, blurring of vision, and occasional injection in one eye [2]. Ocular autoimmune disease is a rare finding with common variable immunodeficiency (CVID), and its manifestations are not commonly recorded or reported. This case report of an ocular disease with CVID forms a touchstone for the potential of other reported cases in the future and in creating a true association between the two disorders. Although the cause is largely unknown, autoimmune and infectious diseases have previously been proposed as potential etiologies of PSS [3]. In this report, we present the first case of PSS in the setting of CVID.

## 2. Case Report

A sixty-two-year-old Caucasian female presented to the emergency room with complaints of acute right-sided vision changes for the past 12 hours. She reported image distortion, blurriness, and loss of central vision with “flashing lights.” She also experienced mild injection of her right conjunctiva and stated that she has experienced episodic blurry vision accompanied by headaches for the past several months.

Significant medical histories included CVID and ulcerative colitis. Her initial immunologic evaluation revealed a low IgG level at 223 mg/dL, normal IgA, and IgM values. The patient was placed on daily sulfamethoxazole-trimethoprim prophylaxis in the setting of recurrent upper respiratory tract infections including sinusitis and bronchitis. The patient failed the prophylactic antibiotic therapy as she continued to have recurrent, persistent sinusitis. The patient was eventually placed on immunoglobulin therapy, and her infections subsided. In addition, the patient has notably complained of intermittent blurry vision in the right eye for several years. Acutely, the patient displayed worsened right eye vision distortion and was evaluated in the emergency room.

In the emergency room, the physical exam was significant for mildly injected right conjunctiva and visual acuity of 20/70 in the right eye and 20/25 in the left eye. The right intraocular pressure (IOP) was measured at 34 mmHg and 12 mmHg in the left. Color vision was intact bilaterally with reactive and equal pupils. No signs of trauma, photophobia, eye drainage, or hyphema were noted. Extraocular eye movements were intact, and no other focal deficits were noted on neurological exam.

The ophthalmology slit lamp examination showed 4-5 trace cells in anterior chamber of right eye confirming anterior chamber uveitis. There were no keratic precipitates, and the iris appeared normal on direct illumination bilaterally. The left eye exam did not show any cells in the anterior chamber or evidence of corneal edema. Gonioscopy and dilated fundus examination were unremarkable. Cup to disc ratio was within normal limits, and no afferent pupillary defects were noted.

The patient was acutely treated with three rounds of dorzolamide/timolol and 0.2% brimonidine with a subsequent decrease in the right eye's IOP to 24 mmHg. She was discharged with brimonidine 0.2% and dorzolamide/timolol two times a day in the right eye. After four days of the initial presentation, a follow-up IOP revealed 41 mmHg of the right and 18 mmHg of the left eye. There was poor foveal reflex in the right eye, and 0-1 cells were noted in the anterior chamber. The patient was diagnosed with Posner-Schlossman syndrome given the clinical findings of elevated right unilateral ocular pressure and anterior segment inflammation with right blurry vision.

## 3. Discussion

PSS typically affects males between the ages of 20 and 50, but advanced ages and female gender have also been reported. Primary open angle glaucoma has been recorded with recurrent attacks of PSS, and treatment is aimed at reducing intraocular pressure and inflammation during and in between episodes [4]. Other studies have proposed initiation of treatment with confirmed optic disc or nerve changes such as ischemic or glaucomatous change; however, this still remains as an ongoing debate. With recurrent attacks as in our case report, the patient was treated as this may have eventually led to optic disc and nerve compromise if left untreated [5, 6].

PSS remains a rare condition with uncertain etiology and no associated systemic conditions. It has been proposed to be linked to autoimmune conditions [7]. A study performed in Japan involving 22 patients with PSS showed HLA-Bw54 positive in nine (approximately 41%) patients implicating a possible CD8 T-cell immunogenic role [8].

Symptoms have been noted to resolve within days to weeks with initial attacks gone unnoticed. In our case, the patient has reported episodic blurry vision in the past during primary care visits. Drug-induced uveitis was initially ruled out since this would cause a bilateral uveitis presentation. Similarly, one case of bilateral uveitis has been reported as a result of IVIG [9], but our patient presents with unilateral findings making IVIG-induced uveitis unlikely. Therefore, these episodes are consistent with the clinical course of PSS. Reoccurrence is expected, but the intervals in between each occurrence have been noted to increase with time.

CVID is a common heterogenetic immunodeficiency found in adulthood [1]. It is associated with many autoimmune disorders including Sjogren's rheumatoid arthritis and colitis. We feel that this ocular disorder represents a rare association in the spectrum of CVID. Some of which have been reported include granulomatous uveitis and conjunctivitis, chronic anterior uveitis, and birdshot retinopathy [10–12]. We predict this ocular manifestation stems from central T-cell regulatory activity with undetermined cell or immune regulatory process. We feel that PSS is of the same nature, and it represents another ocular manifestation in CVID.

Even though this case involving PSS and CVID may sound coincidental, this opinion may be more frequent in other CVID patients seeking ophthalmic evaluations. CVID may set a stage of immunity dysregulation for the manifestation of many autoimmunity disorders including PSS. We describe the first case of PSS in a patient with CVID and hope that the start of this report will lead to other reports bringing a coincidence into an association between PSS and CVID.

## Abbreviations

PSS: Posner-Schlossman syndrome
CVID: Common variable immunodeficiency
OCT: Optical coherence tomography
IOP: Intraocular pressure.
